# Some Useful Sources

**DOI:** 10.1017/S1474746415000561

**Published:** 2016-01

**Authors:** Carmen Lau Clayton, Laura Davies

**Affiliations:** * School of Sociology and Social Policy, University of Leeds E-mail: C.Lau-Clayton@leeds.ac.uk; ** School of Sociology and Social Policy, University of Leeds E-mail: L.Davies@leeds.ac.uk

## Abstract

We have provided here an annotated list of resources, key organisations and programmes that support young fathers and details of data sources for secondary use. The information complements the citations within the individual articles in this themed section.

We have provided here an annotated list of resources, key organisations and programmes that support young fathers and details of data sources for secondary use. The information complements the citations within the individual articles in this themed section.

## All Party Parliamentary Group on Fatherhood

http://www.appgfatherhood.org/

The All Party Parliamentary Group on Fatherhood is an all-party group established to promote the well-being of children by ensuring that legal and policy frameworks reflect the changing nature of family life whilst also ensuring that legislation promotes active and responsible fatherhood.

## The Family Nurse Partnership

http://fnp.nhs.uk/

The Family Nurse Partnerships (FNPs) is a preventive, intensive home visiting programme for vulnerable, first time young mothers, aged nineteen or under where a specially trained family nurse visits the young mother regularly, from early pregnancy until the child is aged two. The service also supports young fathers via the mothers. Based on international evidence, the FNP programme aims to enable young mothers to have a healthy pregnancy, to improve their child's development and health, to plan for their own futures, and to achieve their aspirations. US evidence using three randomised controlled trials indicates that FNPs are effective in terms of children's school readiness, enhanced level of involvement of fathers and reduced incidences of child injuries, neglect and abuse.

## The Fatherhood Institute

www.fatherhoodinstitute.org.uk

The Fatherhood Institute is a think-and-do-tank, and a registered charity which advocates for strong and positive relationships between children and their fathers, and any father figures, while offering support for both parents in their shared role of caring for children. The Fatherhood Institute also collates, participates in and publicises research; lobbies for legal and policy changes; and helps public services, employers and others become more father-inclusive.

## St Michael's Fellowship

http://www.stmichaelsfellowship.org.uk/

St Michael's Fellowship is a registered charity based in Lambeth (London) that works collaboratively with parents and professionals to provide family support. For young parents specifically, St Michael's Fellowship works with young mothers (aged twenty-two and under) and young fathers (up to the age of twenty-four) to offer free advice and support on a range of issues, such as: parenting; relationships; benefits; housing; finding work; confidence building; pregnancy; education and training; finding childcare; sexual health; substance misuse; letter-writing; domestic violence and mediation in difficult relationships.

## Working with Men

http://www.workingwithmen.org/

Working with Men is a London-based charity that undertakes to empower men of all ages by supporting positive male activity, engagement and involvement. Work undertaken is evidence-based and practice-led and recognises that there are multiple masculinities determined by race, class, sexuality, disability, geography, religion and culture. The organisation supports individuals and groups of men and aims to develop strategies to address oppression of all kinds. They focus particularly on the support of young men and run a number of projects for young fathers and fathers to be. Other areas of work include sexual health and mental health, employment education and training and relationship support.

## Young Dads TV

http://youngdads.tv/

Young Dads TV/YDTV (2010‒2013) gives young fathers their own collective voice through the power of the media and the Internet and is also an information service for UK young fathers (below the age of twenty-five), including step-fathers and carers. YoungDads.tv is run by Media for Development and funded by The Monument Trust. The YDTV website includes a digital ‘Dads Map’ which helps young fathers to find local parenting support based on passcode searches The development of the service (and the Young Dads Council) is documented in Scott Colfer, Hannah Turner-Uaandja and Lemar Johnson (2015) ‘Young Dads TV: Digital Voices of Young Fathers’, *Families, Relationships and Societies*, doi: 10.1332/204674315X14352272532048.

## Young Dads Council

http://youngdadscouncil.co.uk/

The idea for a Young Dads Council grew out of the work of Young Dads TV. This is an innovative organisation which empowers young men by supporting them to act as ‘experts by experience’ in London boroughs and beyond. It offers consultation, workshops, outreach services and staff training to organisations who want to develop father inclusive practice. Young fathers who are members of the council are paid the London living wage and work with the media to challenge the stereotypes that exist around young fathers, while also promoting the needs of young fathers in local community services settings.

## Current Research: ESRC Following Young Fathers Study

http://followingfathers.leeds.ac.uk/

The Following Young Fathers project was funded by the Economic and Social Research Council from 2012 to 2015 to research the lived experiences and support needs of young fathers and the services that support them. The project website provides detailed information about the research project, and links to research outputs including conference and seminar presentations, published articles and a one-day dissemination conference in September 2015. In addition, the Knowledge Bank area of the site provides examples of good practice and links to information about specialist services for young fathers.

## Data Sources

www.timescapes.leeds.ac.uk

www.timescapesarchive.leeds.ac.uk

The growing availability of data resources on fathers, including young fathers, has widened the scope for the secondary analysis of existing data. Where these data are longitudinal, there is scope for further follow up studies over time, and to scale up qualitative enquiry by working across thematically related datasets. The Timescapes Archive is a specialist collection of qualitative longitudinal datasets on life course transitions. Data from the Following Young Fathers study and from other studies on the dynamics of fatherhood (for example, the Timescapes Men as Fathers and Working Families studies) are available for re-use, and further datasets, both classic and newly funded, will become available in future.

